# Early Change in the Pan-Immune-Inflammation Value During Neoadjuvant Chemotherapy Is Associated with Pathological Complete Response in HER2-Positive and Triple-Negative Breast Cancer: A Single-Centre Study with Temporal Validation

**DOI:** 10.3390/jcm15218748

**Published:** 2026-11-09

**Authors:** Oguzhan Hakan Topgul, Perya Abbasoglu, Mahmut Zenciroglu, Caglar Kazim Pekuz, Pelin Basim

**Affiliations:** Department of General Surgery, Istanbul Medipol University, 34810 Istanbul, Türkiye

**Keywords:** breast cancer, neoadjuvant chemotherapy, pan-immune-inflammation value, pathological complete response, tumour biomarkers, systemic inflammation

## Abstract

**Background/Objectives:** Pathological complete response (pCR) after neoadjuvant chemotherapy (NAC) is a validated surrogate endpoint in HER2-positive and triple-negative breast cancer. Most work on blood-count-derived inflammatory indices has examined a single pretreatment value rather than change during treatment. We tested whether an early reduction in the pan-immune-inflammation value (PIV) predicts pCR. **Methods:** We studied 272 patients with HER2-positive or triple-negative stage II–III breast cancer treated at one centre: a discovery cohort treated in 2018–2021 (n = 191) and a temporally independent validation cohort treated in 2022–2023 (n = 81). Early ΔPIV was the proportional change between the baseline complete blood count and the count before the second or third cycle. The 30% reduction threshold was fixed before the analysis for clinical interpretability. Reporting followed STROBE and TRIPOD. **Results:** pCR occurred in 157 patients (57.7%). The median early ΔPIV was −0.44 in patients with pCR and −0.11 in those without (*p* < 0.001). The area under the curve was 0.80 (95% CI 0.73–0.86) in the discovery cohort and 0.84 (0.75–0.93) in validation. pCR was reached by 78.6% of patients with a ≥30% early reduction versus 37.6% without it in the discovery cohort, and by 77.8% versus 27.8% in the validation cohort. In the primary three-variable model, the adjusted odds ratio was 6.45 (2.90–14.32; *p* < 0.001) in the discovery cohort and 27.85 (3.93–197.36; *p* < 0.001) in the validation cohort. Adding early PIV reduction to clinicopathologic factors raised the area under the curve from 0.69 to 0.80 (difference 0.111, 95% CI 0.047–0.175; *p* < 0.001). **Conclusions:** Early PIV reduction is associated with pCR and adds information beyond established factors. At 69% sensitivity and 73% specificity it is a candidate adjunct rather than a decision rule, and prospective multicentre validation is required.

## 1. Introduction

Breast cancer is the most frequently diagnosed cancer in women and a leading cause of cancer death [1]. In locally advanced and biologically aggressive disease, particularly HER2-positive and triple-negative tumours, neoadjuvant chemotherapy (NAC) is the standard first step [2,3]. Pathological complete response (pCR), defined as no residual invasive tumour in the breast and axilla, reflects how sensitive the disease is to systemic treatment and is an accepted surrogate endpoint in these two subtypes, where it is both common and prognostically informative [4,5]. It can only be confirmed after several months of treatment and definitive surgery.

Cancer-associated systemic inflammation supports tumour progression, angiogenesis, immune escape and treatment resistance [6,7,8,9]. Peripheral blood cell counts partly mirror this process, and several indices derived from the routine complete blood count (CBC) have been studied as prognostic markers: the neutrophil-to-lymphocyte ratio (NLR) [10,11], the platelet-to-lymphocyte ratio (PLR), the systemic immune-inflammation index (SII) [12] and the pan-immune-inflammation value (PIV) [13]. PIV combines neutrophil, platelet, monocyte and lymphocyte counts, and so samples four cell lineages rather than two. It was introduced in metastatic colorectal cancer [13] and its prognostic value there has since been confirmed in a meta-analysis [14]. In breast cancer, a high pretreatment NLR has been associated with worse survival [11,15,16] and, in triple-negative disease, with a lower chance of responding to NAC [17,18].

Almost all of this work rests on a single measurement taken before treatment. Systemic inflammation is not static, so the early trajectory of these markers may carry information that no baseline value can. Blood-count indices, including their change during treatment, have predicted outcome in lung cancer and melanoma [19,20,21] but have rarely been examined during NAC for breast cancer. The alternative early marker, tumour-infiltrating lymphocytes, does predict pCR [22,23,24,25] but requires tissue and cannot be repeated during treatment.

We therefore examined whether an early fall in PIV during NAC identifies patients who go on to achieve pCR. The primary outcome was pCR, and the primary predictor was an early ≥30% reduction in PIV. The secondary outcomes were the discriminative ability of early ΔPIV measured as a continuous variable; the reproducibility of the association in a temporally independent cohort; the incremental value of early ΔPIV over established clinicopathologic predictors; consistency across the two molecular subtypes; and the performance of related inflammatory indices.

## 2. Materials and Methods

### 2.1. Study Design, Setting, Ethics and Reporting

This retrospective cohort study was conducted at a single tertiary centre. We reviewed consecutive patients with invasive breast cancer who began NAC between January 2018 and December 2023. Patients treated in 2018–2021 formed the discovery cohort and those treated in 2022–2023 the temporally independent validation cohort, with identical eligibility criteria, laboratory definitions and outcome definitions in both. Reporting follows the STROBE statement [26] and, for the prediction-model components, TRIPOD [27,28]. The marker is intended as an adjunct to established predictors during NAC rather than as a stand-alone test, and no treatment decision in this cohort was based on it.

The study was conducted in accordance with the Declaration of Helsinki and approved by the Non-Interventional Clinical Research Ethics Committee of Istanbul Medipol University (approval number 1346). Record review began after approval. The committee waived informed consent because the design was retrospective and the data were anonymised.

### 2.2. Participants

**Figure 1:**
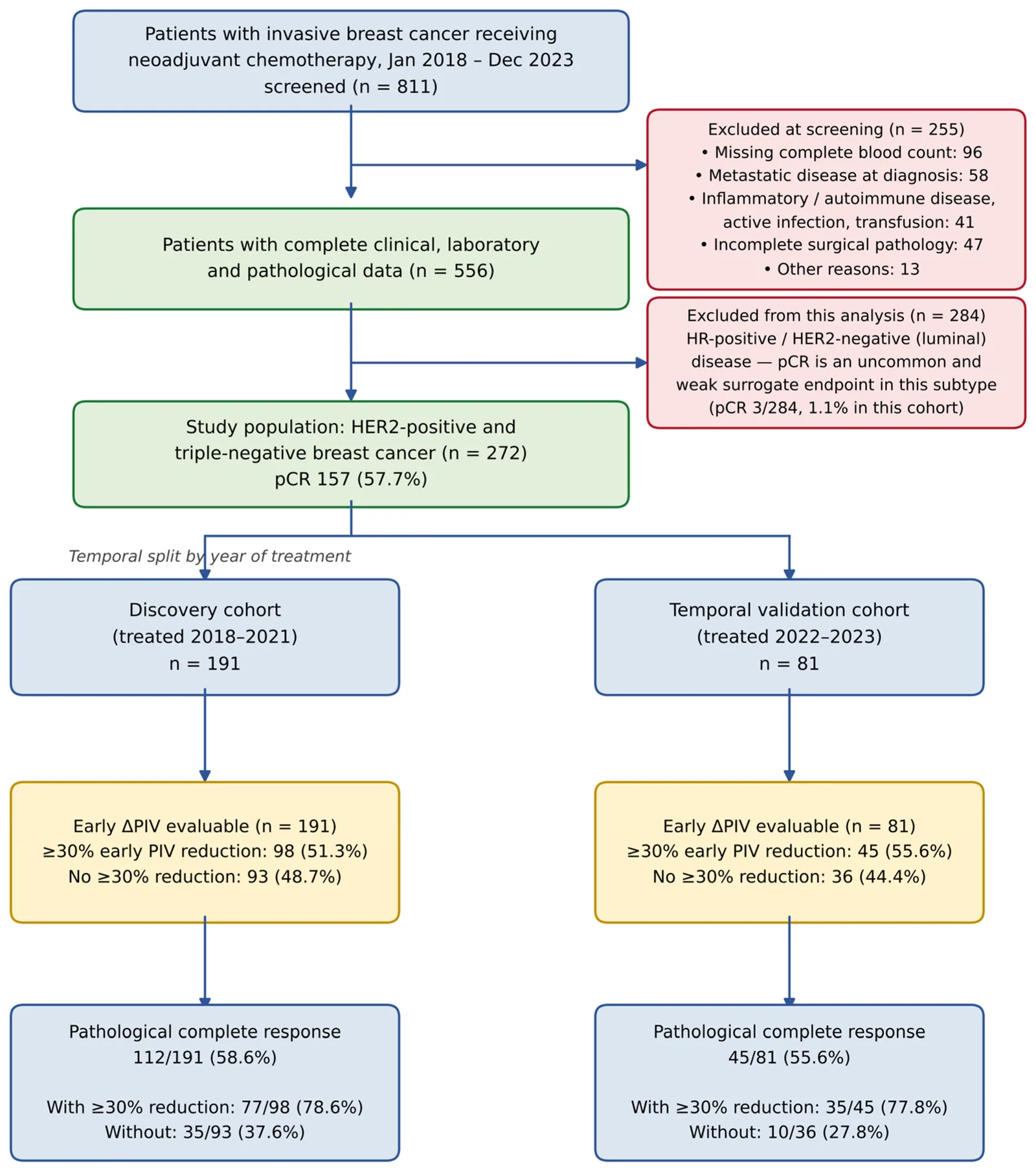
Study flow diagram. Counts are shown for each exclusion reason, for the pre-specified restriction to HER2-positive and triple-negative disease, and for the discovery and temporal validation cohorts, including the number of patients with and without a ≥30% early reduction in the pan-immune-inflammation value (PIV) and the corresponding numbers achieving pathological complete response (pCR).

Eligible patients were at least 18 years of age with histologically confirmed invasive breast carcinoma, clinical stage I–III disease, NAC as first treatment, a complete blood count (CBC) available both before treatment and at the early on-treatment timepoint defined below, and definitive surgical pathology after NAC. Exclusion criteria were metastatic disease at diagnosis, active infection at the time of blood sampling, autoimmune or chronic inflammatory disease, concurrent haematologic malignancy, chronic corticosteroid or immunosuppressive therapy, transfusion within the preceding month, and missing data required for the primary analysis (Figure 1).

The analysis was restricted to HER2-positive and triple-negative tumours, a restriction defined before the analysis and applied at screening, because pCR is a validated surrogate of long-term outcome principally in these subtypes and is uncommon in luminal disease [4]. The excluded luminal patients are compared with the study population in Supplementary Table S5.

### 2.3. Neoadjuvant Treatment

Regimens followed contemporaneous national and institutional guidelines [29] and were chosen by the treating oncologist. Of all 272 patients, 221 (81.3%) received an anthracycline and 252 (92.6%) a taxane. All 119 triple-negative patients received a platinum agent, as did 13 of 153 HER2-positive patients, giving 132 of 272 (48.5%) overall. Dual HER2 blockade with trastuzumab and pertuzumab was given to 133 of the 153 HER2-positive patients (86.9%) and to no triple-negative patient (133 of 272, 48.9%). Dose-dense scheduling was used in 78 patients (28.7%) and granulocyte colony-stimulating factor (G-CSF) support in 107 (39.3%). All patients received the same corticosteroid premedication protocol.

### 2.4. Blood Sampling and Biomarker Definitions

No blood was drawn for research. We used the CBCs obtained in routine care before each chemotherapy cycle and again before surgery; these are recovery counts taken immediately before the next cycle, not nadir counts. Baseline (T0) was before the first cycle, early treatment (T1) before the second or third cycle, and post-neoadjuvant (T2) after NAC and before surgery. Counts drawn during documented infection, hospitalisation or transfusion were not used. The interval in days between T0 and T1 was not recorded in the institutional database and could not be retrieved; this is addressed in the Limitations Section.

Four indices were derived from the neutrophil, lymphocyte, monocyte and platelet counts: NLR = neutrophils/lymphocytes; PLR = platelets/lymphocytes; SII = platelets × neutrophils/lymphocytes; PIV = neutrophils × platelets × monocytes/lymphocytes. The early change in PIV was the proportional difference between T1 and T0 (early ΔPIV = (PIV_T1 − PIV_T0)/PIV_T0), so that a negative value means a fall. The 30% threshold was fixed before the analysis as a round, clinically interpretable cut-off, was not selected to maximise discrimination, and was applied to the validation cohort without recalibration. The threshold maximising the Youden index in the discovery cohort, and the optimism of that data-driven choice, are reported in Supplementary Table S4.

### 2.5. Outcomes

The primary outcome was pCR, defined as the absence of residual invasive carcinoma in the breast and axillary lymph nodes (ypT0/is ypN0) [4,30]; residual ductal carcinoma in situ with negative nodes counted as pCR. The primary predictor was an early ≥30% reduction in PIV. Secondary outcomes were those listed at the end of the Introduction, in that order.

### 2.6. Statistical Analysis

Continuous variables are reported as medians with interquartile ranges and compared with the Mann–Whitney U test; categorical variables are reported as counts with percentages and compared with the chi-square test, or Fisher’s exact test where an expected cell count was below five. The unadjusted biomarker comparisons carry Benjamini–Hochberg false discovery rate q values [31]. Areas under the receiver operating characteristic curve are reported with DeLong confidence intervals and compared with the DeLong test [32]; bootstrap resampling with 2000 replications provided the confidence intervals for the reclassification measures.

The primary model was kept small. With 112 responders and 79 non-responders in the discovery cohort and 45 and 36 in the validation cohort, a seven-covariate model gives about 11 and 5 events per variable [33,34]. We therefore specified, before refitting, a three-variable model containing the early ≥30% PIV reduction, baseline PIV and molecular subtype, which gives 26 and 12 events per variable. Baseline PIV was entered on the natural log scale rather than dichotomised [35], with odds ratios per 10% higher value; the seven-covariate model is retained in Supplementary Table S3, where low baseline PIV is defined by the discovery-cohort median of 375. Because early ΔPIV is calculated from baseline PIV the two are coupled, and regression to the mean is possible [36], so we report their correlation, the variance inflation factors, and two analyses that break the coupling: log T1 PIV adjusted for log baseline PIV, and the component of early ΔPIV orthogonal to baseline PIV. We also analysed the early proportional change in each cell count and decomposed the variance of the log change in PIV across its four components.

Incremental value was assessed by adding the early ≥30% PIV reduction to a model containing molecular subtype, clinical stage, histologic grade and Ki-67, reported as the change in the area under the curve with its confidence interval and DeLong *p* value, the likelihood-ratio test, and the continuous net reclassification and integrated discrimination improvements [37]; apparent discrimination was corrected for optimism by bootstrapping with 500 replications [34]. In the validation cohort the discovery coefficients were applied unchanged, with discrimination, calibration slope, calibration intercept and Brier score reported [38], and a model refitted within that cohort reported separately. Treatment variables were added to the adjustment set in a further sensitivity analysis. Cohort size was fixed by the number of eligible patients, so no sample size calculation was performed; the sample exceeds the minimum suggested for a model of this size [39]. No analysis variable had missing values, so all analyses are complete-case by construction. Tests were two-sided with *p* < 0.05 considered significant; analyses used Python 3.13 with statsmodels 0.15, scipy 1.18 and scikit-learn 1.9.

## 3. Results

### 3.1. Study Population

Of 811 patients screened, 255 were excluded: 96 for missing CBC data, 58 for metastatic disease at diagnosis, 41 for inflammatory or autoimmune disease, active infection or recent transfusion, 47 for incomplete surgical pathology and 13 for other reasons. Of the remaining 556, the 284 with luminal disease were excluded as planned, leaving 272 patients: 191 in the discovery cohort and 81 in the validation cohort (Figure 1). No patient had stage I disease. The excluded luminal patients were similar in age, stage, grade and Ki-67 but had higher baseline PIV and a pCR rate of 1.1% (3 of 284) against 57.7% in the study population (Supplementary Table S5). The two study cohorts were comparable in every baseline characteristic examined (Table 1).

### 3.2. Early PIV Change and Pathological Complete Response

**Figure 2:**
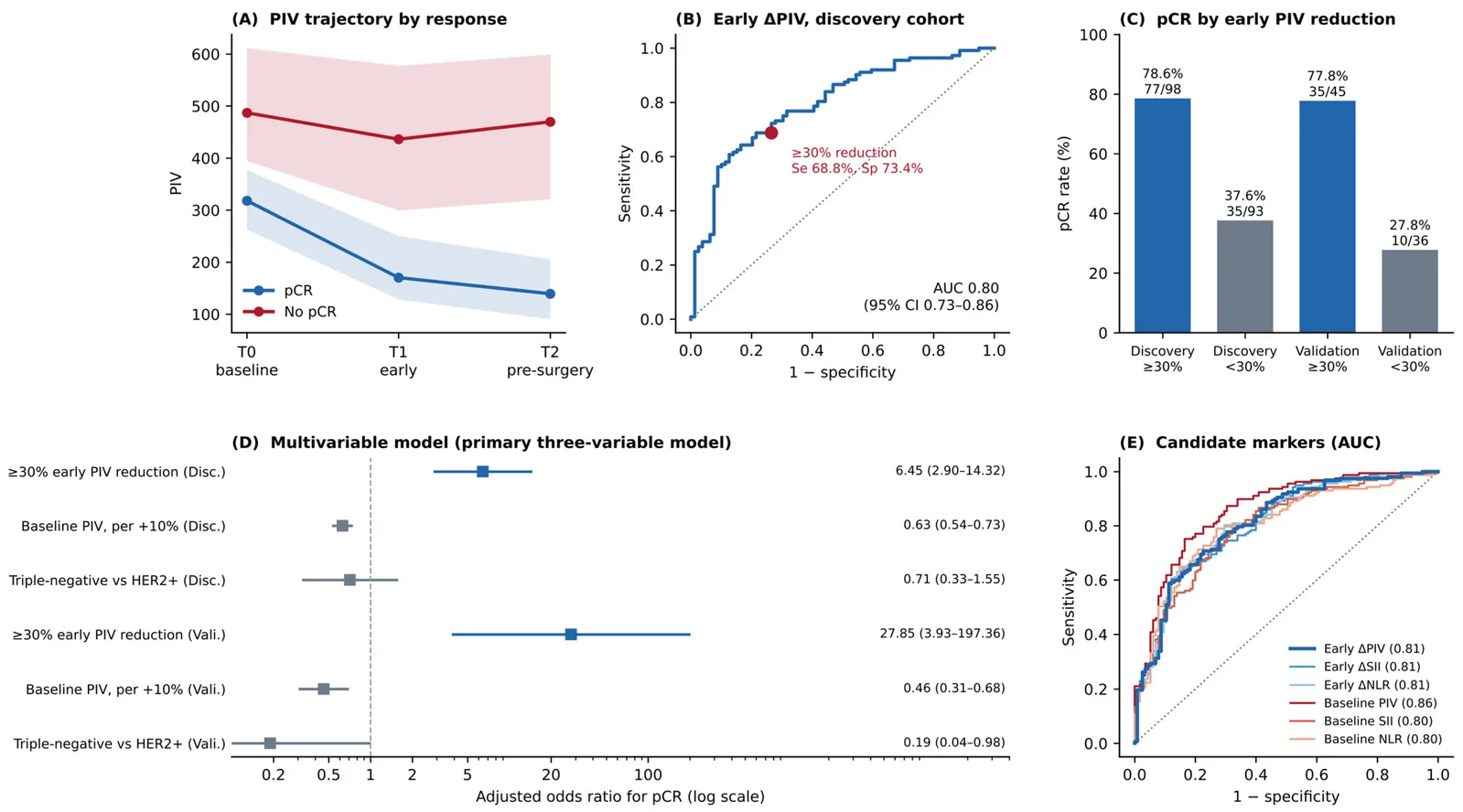
Early change in the pan-immune-inflammation value (PIV) and pathological complete response (pCR). (**A**) Median PIV at baseline (T0), early treatment (T1) and before surgery (T2) by pCR status, with shaded interquartile ranges. (**B**) Receiver operating characteristic curve for early ΔPIV in the discovery cohort, with the pre-specified 30% threshold marked. (**C**) pCR rates with and without a ≥30% early PIV reduction, by cohort; counts are shown above each bar. (**D**) Adjusted odds ratios from the primary three-variable model in both cohorts (log scale). Blue markers indicate the early ≥30% PIV reduction and grey markers the other two covariates; the colours carry no further meaning. (**E**) Receiver operating characteristic curves for six candidate markers in the whole cohort, with areas under the curve in parentheses.

pCR was achieved by 157 of 272 patients (57.7%): 112 of 191 (58.6%) in the discovery cohort and 45 of 81 (55.6%) in validation. Patients who achieved pCR had both a lower baseline PIV and a larger early fall in PIV. The median early ΔPIV was −0.44 (IQR −0.57 to −0.29) with pCR and −0.11 (−0.30 to 0.08) without it (*p* < 0.001). The gap was already present at T1 and persisted to surgery (Figure 2A). Corresponding differences were seen for SII, NLR and PLR (Table 2).

Early ΔPIV, analysed as a continuous variable, discriminated pCR with an area under the curve of 0.80 (95% CI 0.73–0.86) in the discovery cohort and 0.84 (0.75–0.93) in the validation cohort (Figure 2B). At the 30% threshold, sensitivity was 68.8% and specificity was 73.4% in the discovery cohort (positive predictive value 78.6%, negative predictive value 62.4%) and 77.8% and 72.2% in the validation cohort; a data-driven threshold would have been close to the one used and no less optimistic (Supplementary Table S4). An early reduction of at least 30% was present in 98 of 191 discovery patients (51.3%) and 45 of 81 validation patients (55.6%). pCR was reached by 77 of these 98 (78.6%) versus 35 of 93 without a reduction (37.6%) in the discovery cohort, and by 35 of 45 (77.8%) versus 10 of 36 (27.8%) in the validation cohort (both *p* < 0.001; Figure 2C).

### 3.3. Which Blood Count Component Drives the Early Change

The early fall in PIV was not a uniform suppression of all cell lines. Neutrophils fell by a median of 24% in patients who achieved pCR against 6% in those who did not, and monocytes by 16% against 5%; platelets fell by 7% against 2%. Lymphocytes moved in the opposite direction, rising by a median of 8% with pCR and 2% without it (all *p* < 0.001; Table 2). Taken alone, the early change in neutrophils discriminated pCR as well as the composite index (area under the curve 0.81, 95% CI 0.75–0.86 versus 0.81 for early ΔPIV; DeLong *p* = 0.657), followed by monocytes (0.79) and platelets (0.74). In a decomposition of the variance of the log change in PIV, neutrophils accounted for 47%, monocytes 29%, platelets 13% and lymphocytes 12%.

### 3.4. Independent Association and Temporal Validation

In the primary three-variable model, an early ≥30% reduction in PIV was independently associated with pCR in the discovery cohort (adjusted odds ratio 6.45, 95% CI 2.90–14.32; *p* < 0.001), together with a lower baseline PIV (odds ratio 0.63 per 10% higher baseline value, 0.54–0.73; *p* < 0.001). The same model in the validation cohort gave an adjusted odds ratio of 27.85 (3.93–197.36; *p* < 0.001) for the early reduction (Table 3, Figure 2D). Analysed continuously, each additional 10% of early reduction was associated with an adjusted odds ratio of 1.61 (1.33–1.94; *p* < 0.001) in discovery and 2.36 (1.49–3.73; *p* < 0.001) in validation. The width of the validation intervals reflects 45 responders and 36 non-responders and shows direction rather than magnitude.

**Figure 3:**
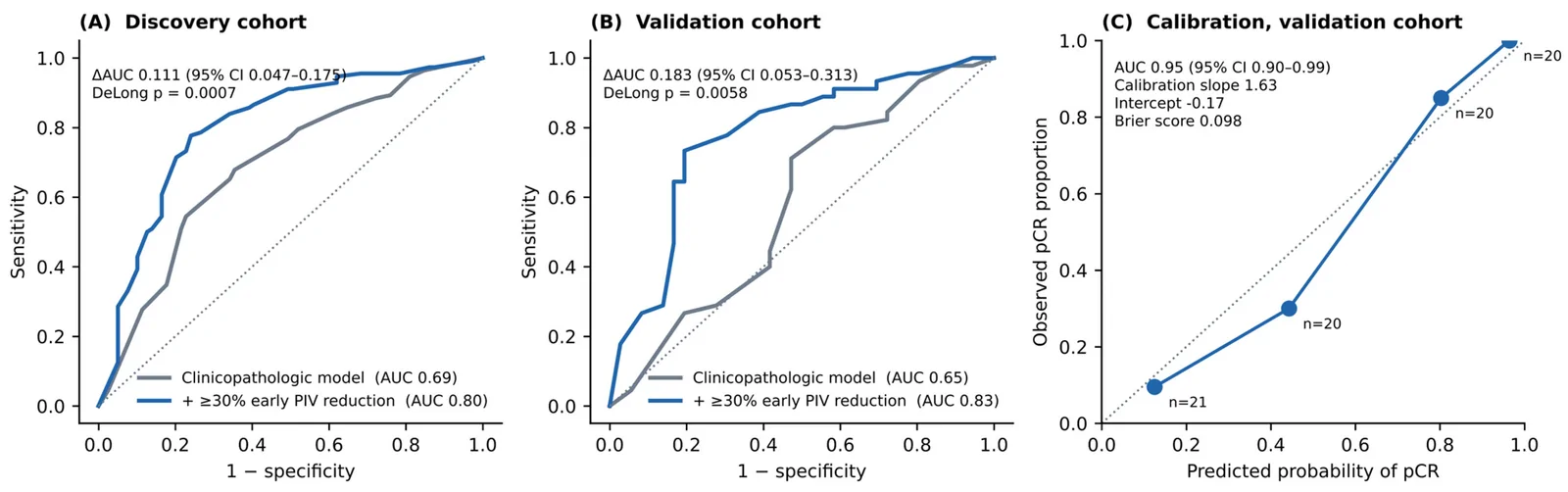
Incremental value of an early ≥30% PIV reduction over established clinicopathologic factors. (**A**) Discovery cohort and (**B**) validation cohort: receiver operating characteristic curves for a model containing molecular subtype, clinical stage, histologic grade and Ki-67, with and without the early PIV reduction. (**C**) Calibration of the primary three-variable discovery model applied to the validation cohort without recalibration, by quartile of predicted probability; the dotted line is perfect calibration.

Applied to the validation cohort with its coefficients unchanged, the discovery model gave an area under the curve of 0.95 (0.90–0.99), a calibration slope of 1.63 (0.89–2.37), an intercept of −0.17 and a Brier score of 0.098 (Table 3, Figure 3C); optimism correction reduced the apparent discovery figure from 0.88 to 0.87.

Baseline PIV and early ΔPIV were positively correlated (Spearman ρ = 0.31, *p* < 0.001): a higher baseline PIV went with a smaller early fall, the opposite of the pattern regression to the mean would produce. Variance inflation factors were all below 1.2. Removing the arithmetic coupling changed nothing: with log T1 PIV modelled against log baseline PIV the adjusted odds ratio was 0.73 per 10% higher T1 PIV (0.64–0.84), and using the component of early ΔPIV orthogonal to baseline PIV it was 1.49 per 10% greater baseline-independent reduction (1.25–1.77; both *p* < 0.001).

### 3.5. Incremental Value

A model containing molecular subtype, clinical stage, histologic grade and Ki-67 discriminated pCR with an area under the curve of 0.69 (95% CI 0.61–0.77) in the discovery cohort; adding the early ≥30% PIV reduction raised this to 0.80 (0.74–0.87), a difference of 0.111 (0.047–0.175; DeLong *p* < 0.001; likelihood-ratio χ^2^ = 32.6, *p* < 0.001), with a continuous net reclassification improvement of 0.84 (0.59–1.10) and an integrated discrimination improvement of 0.158 (0.070–0.269). In the validation cohort the area under the curve rose from 0.65 (0.52–0.77) to 0.83 (0.74–0.92), a difference of 0.183 (0.053–0.313; *p* = 0.006) (Figure 3A,B). After correction for optimism the discovery figures were 0.67 and 0.79.

### 3.6. Subtypes and Other Inflammatory Markers

The association held in both tumour types. Among the 153 HER2-positive patients, pCR was reached by 68 of 83 (81.9%) with an early reduction versus 31 of 70 (44.3%) without it (odds ratio 5.70, 95% CI 2.74–11.85); among the 119 triple-negative patients, by 44 of 60 (73.3%) versus 14 of 59 (23.7%) (odds ratio 8.84, 3.86–20.25). The test for interaction was not significant (*p* = 0.200), so the data do not show a difference between subtypes, though with these numbers they would not detect a modest one (Supplementary Table S1).

Early ΔPIV was not better than the related indices. In the whole cohort the area under the curve was 0.81 for early ΔPIV, early ΔSII and early ΔNLR alike, and neither comparison with early ΔPIV reached significance on the DeLong test (both *p* > 0.44). Baseline PIV gave the numerically highest value, 0.86, but the difference from early ΔPIV was not significant (−0.048, 95% CI −0.117 to 0.021; *p* = 0.176). A ≥30% early reduction in SII was accompanied by pCR in 95 of 111 patients (85.6%) versus 62 of 161 (38.5%). These markers are strongly inter-correlated and the data cannot separate them (Figure 2E, Supplementary Table S4). We report PIV as the primary measure because it was specified in advance, not because it outperformed the others.

### 3.7. Sensitivity Analyses

The signal was not explained by treatment-induced cytopenia or growth-factor support. Severe cytopenia at T1 was rare, as expected for counts taken before the next cycle rather than at nadir: across the whole cohort the median T1 neutrophil count was 3.26 × 10^9^/L (IQR 2.48–4.49) and the median T1 lymphocyte count 1.61 × 10^9^/L (1.37–1.84), and one discovery patient had a T1 neutrophil count below 1.0 × 10^9^/L. Against a reference estimate of 8.73 (3.78–20.19) in the seven-covariate model, the adjusted odds ratio for the early reduction ranged from 7.95 to 9.28 across six sensitivity analyses covering G-CSF exclusion and adjustment, three definitions of low T1 counts, and the addition of the treatment variables to the adjustment set (Supplementary Table S2). G-CSF use was distributed similarly between patients who did and did not achieve pCR (*p* = 0.693) and between those with and without an early reduction (*p* = 0.379). Every patient received the same corticosteroid premedication, so its effect cannot be separated from the treatment effect here.

## 4. Discussion

In this single-centre study with temporal validation, an early fall in PIV during NAC was associated with pCR in HER2-positive and triple-negative breast cancer. The association survived adjustment for established clinicopathologic predictors, was reproduced in a cohort treated in later years, and added discrimination to a model built from subtype, stage, grade and Ki-67.

What distinguishes this analysis from earlier work on blood-count indices in breast cancer is the use of change rather than a single value. Previous studies examined pretreatment NLR, PLR or SII as fixed predictors of survival or response [11,15,16,17,18]. A baseline value describes the host–tumour relationship at diagnosis; it cannot describe what the tumour does once treatment starts. Both carry information and neither replaces the other: baseline PIV gave the numerically highest area under the curve of any single marker, early ΔPIV remained associated with pCR after adjusting for it, and the two were positively rather than negatively correlated. Early ΔPIV was not superior to early ΔSII or early ΔNLR, and with 272 patients the differences between these closely related indices sit well inside the confidence intervals.

The component analysis helps explain what the index measures. The early fall in PIV was driven mainly by falling neutrophils and monocytes while lymphocytes rose. Generalised marrow suppression would lower all four lineages, including lymphocytes, so this pattern is not simply a chemotherapy toxicity signal. It is more consistent with a shift away from the myeloid-dominated inflammatory state that accompanies an active tumour [6,7,9]. Neutrophils can promote tumour growth through proangiogenic mediators and proteases, monocytes can become tumour-associated macrophages that suppress immunity, platelets can shield circulating tumour cells, and lymphocytes carry the antitumour response [6,8,22]. The early change in neutrophils alone performed as well as the four-component index, so PIV’s value here lies in convenience rather than in any demonstrated advantage of combining four counts. Tumour-infiltrating lymphocytes also predict pCR in both subtypes studied here [22,23,24,25], but they require a biopsy and cannot be repeated through treatment, whereas PIV comes from a test already performed before every cycle.

The clinical reading should stay modest. Adding the early PIV reduction raised the area under the curve by 0.111, a larger gain than is usually reported when a blood-based marker is added to a clinical model. A change in discrimination of this size still does not establish clinical usefulness on its own: what matters is whether the resulting probabilities would change a decision, and at this operating point they would not. At the 30% threshold, sensitivity was 69% and specificity was 73%, so roughly one in three patients who eventually achieve pCR would show no early reduction, and the marker cannot be used to withhold or change treatment in an individual patient. What the data support is the weaker claim that early inflammatory kinetics carry information beyond the standard clinicopathologic variables, enough to justify prospective evaluation rather than adoption into practice.

### Limitations

The design was retrospective, so selection bias and residual confounding cannot be excluded. Patients were excluded at screening mainly for missing blood counts or incomplete pathology, and those patients could not be characterised from the available records; if incomplete records were more common in patients treated irregularly, the analysed cohort is likely to be healthier than the source population and the performance reported here optimistic.

The timing of T1 is the clearest weakness. T1 was the count before the second or third cycle, and regimens were given every two or three weeks, so the interval between T0 and T1 varied between patients. Sampling dates were not recorded and could not be reconstructed, so we cannot report the median interval or adjust for it. Variable timing would be expected to add noise and bias the association towards the null rather than create it, but it limits the precision of any threshold and must be fixed prospectively. Several variables that would normally appear in a breast cancer baseline table were also unavailable: separate oestrogen and progesterone receptor status within the hormone receptor-negative groups, menopausal status, tumour size and separate clinical T and N categories. Residual confounding by tumour burden in particular cannot be excluded.

The validation cohort was small, with 45 responders and 36 non-responders, so its confidence intervals are wide and its refitted estimates unstable; this is why the primary model was restricted to three covariates and why validation relies on the transported discovery model. Validation was temporal and within one centre, so it tests reproducibility over time but not transportability to other populations, laboratories or analysers, and PIV is analyser-sensitive. All patients received the same corticosteroid premedication; steroids substantially affect neutrophil and lymphocyte counts, and with no unexposed group this effect could not be examined. Being shared by every patient it cannot explain the difference between responders and non-responders, but it may shift the absolute value of the threshold in cohorts premedicated differently. Finally, the endpoint was pCR; no survival data are presented, so whether early PIV change predicts long-term outcome is unknown.

## 5. Conclusions

An early reduction in the pan-immune-inflammation value during neoadjuvant chemotherapy was associated with pathological complete response in HER2-positive and triple-negative breast cancer, and added discrimination beyond subtype, stage, grade and Ki-67 in both a discovery and a temporally independent validation cohort. The index is calculated from a blood count that is already taken before every cycle, which makes repeated measurement free. At 69% sensitivity and 73% specificity it is a candidate adjunct rather than a decision rule, too modest to guide treatment in an individual patient, and it did not outperform the related SII and NLR indices. Prospective multicentre studies with fixed sampling intervals and survival follow-up are needed before early inflammatory kinetics can be used in response-adapted care.

## Figures and Tables

**Table 1:** Baseline characteristics of the discovery and validation cohorts.

Characteristic	Discovery (n = 191)	Validation (n = 81)	*p* Value
***Demographics***			
Age, years	47 (41–54)	46 (40–55)	0.927
***Tumour characteristics***			
Clinical stage II	113 (59.2)	53 (65.4)	0.404
Clinical stage III	78 (40.8)	28 (34.6)	0.404
HER2-positive	105 (55.0)	48 (59.3)	0.605
Triple-negative	86 (45.0)	33 (40.7)	0.605
Histologic grade 1	36 (18.8)	13 (16.0)	0.706
Histologic grade 2	38 (19.9)	17 (21.0)	0.968
Histologic grade 3	117 (61.3)	51 (63.0)	0.898
Ki-67, %	38.0 (30.1–46.4)	40.2 (32.0–48.7)	0.120
***Inflammatory markers***			
Baseline PIV	375 (296–481)	388 (293–508)	0.887
Baseline SII	630 (459–840)	594 (423–876)	0.664
Baseline NLR	2.57 (2.19–3.14)	2.64 (2.13–3.15)	0.697
Baseline PLR	159 (133–189)	161 (128–186)	0.726
T1 PIV	258 (159–374)	246 (148–436)	0.913
Early ΔPIV, proportional	−0.31 (−0.50 to −0.13)	−0.33 (−0.52 to −0.08)	0.659
Early ΔSII, proportional	−0.23 (−0.39 to −0.08)	−0.25 (−0.40 to −0.03)	0.690
≥30% early PIV reduction	98 (51.3)	45 (55.6)	0.611
***Neoadjuvant treatment***			
Anthracycline-based	160 (83.8)	61 (75.3)	0.143
Taxane-based	174 (91.1)	78 (96.3)	0.212
Platinum, triple-negative patients only	86/86 (100)	33/33 (100)	1.000
Dual HER2 blockade, HER2-positive patients only	92/105 (87.6)	41/48 (85.4)	0.907
Dose-dense schedule	59 (30.9)	19 (23.5)	0.274
G-CSF support	73 (38.2)	34 (42.0)	0.657
***Outcome***			
Pathological complete response	112 (58.6)	45 (55.6)	0.736

Values are median (interquartile range) or n (%). Percentages refer to the whole cohort unless a denominator is given. *p* values from the Mann–Whitney U test for continuous variables and the chi-square test, or Fisher’s exact test where expected counts were below five, for categorical variables. G-CSF, granulocyte colony-stimulating factor; NLR, neutrophil-to-lymphocyte ratio; PIV, pan-immune-inflammation value; PLR, platelet-to-lymphocyte ratio; SII, systemic immune-inflammation index; T1, early on-treatment timepoint; ΔPIV, proportional change in PIV between baseline and T1.

**Table 2:** Inflammatory indices and early changes in individual blood counts according to pathological complete response, whole cohort.

Variable	pCR (n = 157)	No pCR (n = 115)	*p* Value	q Value
***Baseline indices***				
Baseline PIV	318 (263–377)	487 (395–611)	<0.001	<0.001
Baseline SII	497 (366–679)	815 (624–1029)	<0.001	<0.001
Baseline NLR	2.30 (2.02–2.65)	3.04 (2.62–3.66)	<0.001	<0.001
Baseline PLR	140 (121–174)	182 (156–210)	<0.001	<0.001
***Early on-treatment indices***				
T1 PIV	170 (129–250)	436 (300–578)	<0.001	<0.001
Early ΔPIV, proportional	−0.44 (−0.57 to −0.29)	−0.11 (−0.30 to 0.08)	<0.001	<0.001
Early ΔSII, proportional	−0.35 (−0.44 to −0.20)	−0.08 (−0.23 to 0.07)	<0.001	<0.001
Early ΔNLR, proportional	−0.30 (−0.38 to −0.18)	−0.07 (−0.20 to 0.04)	<0.001	<0.001
Early ΔPLR, proportional	−0.13 (−0.20 to −0.06)	−0.03 (−0.10 to 0.03)	<0.001	<0.001
***Early proportional change in each blood count***				
Neutrophils	−0.24 (−0.32 to −0.14)	−0.06 (−0.16 to 0.04)	<0.001	<0.001
Lymphocytes	0.08 (0.03 to 0.12)	0.02 (−0.02 to 0.06)	<0.001	<0.001
Monocytes	−0.16 (−0.21 to −0.10)	−0.05 (−0.11 to 0.01)	<0.001	<0.001
Platelets	−0.07 (−0.10 to −0.03)	−0.02 (−0.05 to 0.02)	<0.001	<0.001

Values are medians (interquartile range). The *p* values from the Mann–Whitney U test; q values are Benjamini–Hochberg false discovery rate adjusted *p* values across the 13 comparisons in this table. A negative proportional change indicates a fall between baseline and T1. Abbreviations as in Table 1; pCR, pathological complete response.

**Table 3:** Primary multivariable model for pathological complete response and performance of the discovery model in the validation cohort.

	Discovery (n = 191)	*p* Value	Validation (n = 81)	*p* Value
***Primary three-variable model, odds ratio (95% CI)***				
≥30% early PIV reduction	6.45 (2.90–14.32)	<0.001	27.85 (3.93–197.36)	<0.001
Baseline PIV, per 10% higher	0.63 (0.54–0.73)	<0.001	0.46 (0.31–0.68)	<0.001
Triple-negative vs. HER2-positive	0.71 (0.33–1.55)	0.391	0.19 (0.04–0.98)	0.047
***Early ΔPIV analysed continuously, odds ratio (95% CI)***				
Per additional 10% early reduction	1.61 (1.33–1.94)	<0.001	2.36 (1.49–3.73)	<0.001
***Events per variable***				
Three-variable model	26.3	—	12.0	—
Seven-covariate model (Table S3)	11.3	—	5.1	—
***Performance of the discovery model applied to the validation cohort without recalibration***				
Area under the curve (95% CI)	0.88 (0.83–0.93) *	—	0.95 (0.90–0.99)	—
Optimism-corrected area under the curve	0.87	—	not applicable	—
Calibration slope (95% CI)	1 by construction	—	1.63 (0.89–2.37)	—
Calibration intercept	0 by construction	—	−0.17	—
Brier score	—	—	0.098	—

* Apparent discrimination of the three-variable model in the cohort in which it was fitted. Odds ratios in the validation column come from the same three-variable model refitted within the validation cohort and are reported for direction rather than magnitude, because that cohort contains 45 responders and 36 non-responders. The performance rows report the discovery model applied to the validation cohort with its coefficients unchanged. CI, confidence interval; other abbreviations as in Table 1 and Table 2.

## Data Availability

The de-identified dataset analysed in this study is available from the corresponding author on reasonable request, subject to institutional data-sharing policies and ethics approval.

## Supplementary Materials

The following supporting information can be downloaded at: https://www.mdpi.com/article/10.3390/jcm15218748/s1, Table S1, Pathological complete response according to early PIV reduction, by molecular subtype; Table S2, Sensitivity analyses for the association between a ≥30% early PIV reduction and pCR (discovery cohort), and distribution of blood counts at the early timepoint; Table S3, Seven-covariate multivariable model (sensitivity analysis); Table S4, Pairwise comparison of candidate markers for predicting pCR (whole cohort, DeLong test); Table S5, Study population compared with the hormone receptor-positive, HER2-negative patients excluded by design.
