# Supplementary material for: Early Change in the Pan-Immune-Inflammation Value During Neoadjuvant Chemotherapy Is Associated with Pathological Complete Response in HER2-Positive and Triple-Negative Breast Cancer: A Single-Centre Study with Temporal Validation
Source: J Clin Med. 2026 Nov 9;15(21):8748. doi: 10.3390/jcm15218748 (PMC13764517; doi:10.3390/jcm15218748)
Supplement: Supplementary file 1 [file jcm-15-08748-s001.zip › jcm-4630993-supplementary.pdf]

Supplementary Materials

Supplementary Materials

Early Change in the Pan-Immune-Inflammation Value During Neoadjuvant Chemotherapy Is Associated with Pathological Complete Response in HER2-Positive and Triple-Negative Breast Cancer: A Single-Centre Study with Temporal Validation

Topgul OH, Abbasoglu P, Zenciroglu M, Pekuz CK, Basim P

Table S1. Pathological complete response according to early PIV reduction, by molecular subtype

Subtype / cohort	≥30% early PIV reduction, pCR/n (%)	No ≥30% reduction, pCR/n (%)	Odds ratio (95% CI)
**HER2-positive (n = 153)**	68/83 (81.9)	31/70 (44.3)	5.70 (2.74–11.85)
— Discovery	43/55 (78.2)	24/50 (48.0)	—
— Validation	25/28 (89.3)	7/20 (35.0)	—
**Triple-negative (n = 119)**	44/60 (73.3)	14/59 (23.7)	8.84 (3.86–20.25)
— Discovery	34/43 (79.1)	11/43 (25.6)	—
— Validation	10/17 (58.8)	3/16 (18.8)	—

Odds ratios are unadjusted and are shown for the pooled cohort only, because the per-cohort subgroups contain too few events to support stable estimates. Adjusted for clinical stage, histologic grade, Ki-67 and baseline PIV, the odds ratio was 5.89 (2.46–14.12; $p < 0.001$) in HER2-positive disease and 55.34 (6.87–445.89; $p < 0.001$) in triple-negative disease; the triple-negative estimate rests on 14 events in the reference group and its magnitude should not be interpreted. The test for interaction between early PIV reduction and subtype was not significant ($p = 0.200$). Area under the curve for early ΔPIV was 0.79 (0.71–0.87) in HER2-positive and 0.83 (0.76–0.90) in triple-negative disease.

CI, confidence interval; pCR, pathological complete response; PIV, pan-immune-inflammation value.

Table S2. Sensitivity analyses for the association between a ≥30% early PIV reduction and pCR (discovery cohort), and distribution of blood counts at the early timepoint

Analysis	n	Adjusted odds ratio (95% CI)	p value
Seven-covariate model of Table S3 (reference for these analyses)	191	8.73 (3.78–20.19)	<0.001
Excluding patients who received G-CSF	118	8.56 (2.91–25.17)	<0.001
G-CSF support entered in the model	191	8.86 (3.82–20.54)	<0.001
Excluding T1 neutrophils <1.5 $\times 10^9$/L (n = 11 excluded)	180	7.95 (3.41–18.52)	<0.001
Excluding T1 neutrophils <1.0 $\times 10^9$/L or lymphocytes <0.5 $\times 10^9$/L (n = 1 excluded)	190	8.70 (3.76–20.12)	<0.001
Excluding T1 platelets <100 $\times 10^9$/L (n = 6 excluded)	185	8.47 (3.65–19.62)	<0.001
Adding platinum, dual HER2 blockade, anthracycline, dose-dense scheduling and G-CSF to the adjustment set	191	9.28 (3.93–21.88)	<0.001

All models in this table adjust for molecular subtype, clinical stage, histologic grade, Ki-67, low baseline PIV and age, so that each estimate is comparable with the reference row. The primary analysis of the manuscript is the three-variable model of Table 3, whose odds ratio is 6.45 (2.90–14.32).

Distribution of blood counts at the early timepoint (whole cohort, n = 272; median and interquartile range): neutrophils 3.26 (2.48–4.49) $\times 10^9$/L, minimum 0.65; lymphocytes 1.61 (1.37–1.84) $\times 10^9$/L, minimum 0.85; monocytes 0.55 (0.39–0.80) $\times 10^9$/L; platelets 233 (178–277) $\times 10^9$/L, minimum 80. Corresponding baseline values were neutrophils 3.96 (3.14–4.98), lymphocytes 1.51 (1.32–1.74), monocytes 0.61 (0.43–0.90) and platelets 242 (187–289) $\times 10^9$/L. Counts at T1 are taken immediately before the next cycle and are therefore recovery counts rather than nadir counts, which is why severe cytopenia is rare.

Balance of G-CSF support: G-CSF use did not differ between patients who did and did not achieve pCR (p = 0.693) or between those with and without a ≥30% early PIV reduction (p = 0.379).

All 272 patients received the same corticosteroid premedication protocol, so the effect of steroids on neutrophil and lymphocyte counts cannot be separated from the treatment effect in this cohort.

G-CSF, granulocyte colony-stimulating factor; T1, early on-treatment timepoint.

Table S3. Seven-covariate multivariable model (sensitivity analysis)

Variable	Discovery OR (95% CI)	p	Validation OR (95% CI)	p
≥30% early PIV reduction	8.73 (3.78–20.19)	<0.001	44.73 (5.84–342.47)	<0.001
Low baseline PIV (<375)	13.21 (5.61–31.10)	<0.001	67.18 (8.57–526.57)	<0.001
Triple-negative vs HER2-positive	0.73 (0.33–1.62)	0.440	0.39 (0.09–1.74)	0.217
Clinical stage III	0.47 (0.21–1.05)	0.066	0.33 (0.06–1.71)	0.186
Histologic grade 3	2.23 (0.99–5.04)	0.053	1.37 (0.29–6.43)	0.689
Ki-67 >30%	1.18 (0.49–2.88)	0.711	2.30 (0.34–15.80)	0.397
Age, per year	1.02 (0.98–1.07)	0.340	0.90 (0.82–0.99)	0.027

This model is retained only for comparison with the original submission. The cut-point for low baseline PIV is the discovery-cohort median of 375, now stated explicitly. With seven covariates the model gives about 11 events per variable in the discovery cohort and about 5 in the validation cohort; the validation estimates are correspondingly unstable, as the confidence intervals show, and the pre-specified three-variable model in Table 3 of the main text is the primary analysis. Ridge-penalised estimates in the validation cohort shrink the odds ratio for the early PIV reduction from 44.73 to 6.95, and that for low baseline PIV from 67.18 to 9.69, confirming that the unpenalised magnitudes are inflated while the direction of effect is unchanged.

Table S4. Pairwise comparison of candidate markers for predicting pCR (whole cohort, DeLong test)

Comparison	AUC, early ΔPIV	AUC, comparator	Difference (95% CI)	p value
Early ΔPIV vs early ΔSII	0.811	0.807	0.004 (−0.006 to 0.014)	0.449
Early ΔPIV vs early ΔNLR	0.811	0.809	0.001 (−0.011 to 0.014)	0.824
Early ΔPIV vs baseline PIV	0.811	0.858	−0.048 (−0.117 to 0.021)	0.176
Early ΔPIV vs baseline SII	0.811	0.796	0.014 (−0.057 to 0.086)	0.696
Early ΔPIV vs baseline NLR	0.811	0.802	0.009 (−0.065 to 0.082)	0.814

No comparison reached statistical significance. Areas under the curve by cohort: early ΔPIV 0.80 (discovery) and 0.84 (validation); early ΔSII 0.79 and 0.83; early ΔNLR 0.79 and 0.84; baseline PIV 0.84 and 0.89.

Threshold selection. The 30% threshold used throughout was fixed before the analysis for clinical interpretability and was not chosen to maximise discrimination. For comparison, the threshold maximising the Youden index in the discovery cohort was a 36% reduction, close to the value used but with a different operating point (sensitivity 60.7%, specificity 87.3% against 68.8% and 73.4% at 30%). Bootstrapping with 2000 replications showed that choosing the cut-point from the data in this way is optimistic by 0.030 Youden index units, so a data-driven threshold would have performed no better than the pre-specified one once that optimism is taken into account.

AUC, area under the receiver operating characteristic curve; NLR, neutrophil-to-lymphocyte ratio; SII, systemic immune-inflammation index.

Table S5. Study population compared with the hormone receptor-positive, HER2-negative patients excluded by design

Characteristic	Study population (n = 272)	Luminal, excluded (n = 284)	p value
Age, years	47 (41–54)	49 (42–55)	0.197
Ki-67, %	38.2 (30.6–47.4)	40.0 (30.6–49.8)	0.263
Clinical stage III	106 (39.0)	132 (46.5)	0.089
Histologic grade 3	168 (61.8)	182 (64.1)	0.632
Baseline PIV	377 (295–483)	479 (391–598)	<0.001
Pathological complete response	157 (57.7)	3 (1.1)	<0.001

Values are median (interquartile range) or n (%). The pCR rate of 1.1% in the luminal group is the reason pCR is not an interpretable endpoint in that subtype and the reason these patients were excluded before the analysis.

The 255 patients excluded at the screening stage (Figure 1) could not be characterised, because the records that were incomplete for the analysis were also incomplete for a baseline comparison. This is stated as a limitation in the main text.
